# Risk factors for development of non-specific musculoskeletal pain in preteens and early adolescents: a prospective 1-year follow-up study

**DOI:** 10.1186/1471-2474-8-46

**Published:** 2007-05-23

**Authors:** Ashraf El-Metwally, Jouko J Salminen, Anssi Auvinen, Gary Macfarlane, Marja Mikkelsson

**Affiliations:** 1 Department of Physical and Rehabilitation Medicine, The Rheumatism Foundation Hospital, Pikijärventie 1, 18120 Heinola, Finland; 2 Epidemiology Group, Department of Public Health, University of Aberdeen, Foresterhill, Aberdeen, AB25 2ZD, UK; 3 Tampere School of Public Health, FIN-33014 University of Tampere, Tampere, Finland; 4 Department of Physical and Rehabilitation Medicine, University Hospital of Turku, P.O box 52, 20520 Turku, Finland; 5 Pediatric Research Center, Tampere University Hospital, FIN-33014, Tampere, Finland

## Abstract

**Background:**

Musculoskeletal pain symptoms are common in children and adolescents. These symptoms have a negative impact on children's physical and emotional well-being, but their underlying aetiology and risk factors are still poorly understood. Most of the previous cohort studies were conducted among mid and/or late adolescents and were mainly focused on a specific pain location (e.g. low back pain or neck pain). The purpose of this study is to estimate occurrence of new-onset pain symptoms, in all musculoskeletal locations, in preteens and early adolescents and investigate risk factors for development of these symptoms.

**Methods:**

1756 schoolchildren (mean age 10.8) were recruited from schools in southern Finland. Information was extracted as to whether they experienced musculoskeletal pain and a total of 1192 children were identified as free of musculoskeletal pain symptoms. Information was collected on factors which could potentially predict the development of musculoskeletal pain: headache, abdominal pain, sadness/feeling down, day-time tiredness, difficulty in falling asleep, waking up during nights, level of physical activity and hypermobility. These children were followed-up 1-year later and those with new episodes of non-traumatic and traumatic musculoskeletal pain symptoms were identified.

**Results:**

A total of 1113 schoolchildren (93% of baseline pain-free children) were found at one-year follow-up. New episodes of musculoskeletal pain were reported by 21.5% of these children. Of them 19.4% reported non-traumatic pain and 4.0% reported traumatic pain. The neck was the most commonly reported site with non-traumatic pain, while the lower limb was the most common site for traumatic pain. The independent risk factors for non-traumatic musculoskeletal pain were headache (OR = 1.68, [95% CI 1.16–2.44]) and day-time tiredness (OR = 1.53, [95% CI 1.03–2.26]). The risk factors for traumatic musculoskeletal pain were vigorous exercise (OR = 3.40 [95% CI 1.39–8.31]) and day-time tiredness (OR = 2.97 [95% CI 1.41–6.26]).

**Conclusion:**

This study highlights that there may be two types of pain entities with both distinct and common aspects of aetiology. For primary prevention purposes, school healthcare professionals should pay attention to preteens and early adolescents practicing vigorous exercise (predictor of traumatic pain), reporting headache (predictor of non-traumatic pain) and reporting day-time tiredness (predictor of both types of pain).

## Background

Musculoskeletal pain is a commonly reported symptom in adolescents. Estimates of prevalence vary according to age, case definition of pain, and method of data collection, but in early adolescents as many as 53% may have experienced musculoskeletal pains at some time in their lives [1] and about 15% have persistent musculoskeletal pain of at least once a week [2]. These pain symptoms have a negative impact on children's physical and emotional well-being [3,4] but their underlying aetiology and risk factors are still poorly understood.

Compared to cross-sectional (prevalence) surveys, there have been a relatively small number of cohort (incidence) studies about musculoskeletal pain in children and adolescents. Most of these studies have focused specifically on low back pain, and have reported an incidence proportion ranging from 12% to 22% [5-7], depending on the age of the children and the period between the initial and the follow-up assessments. A recent prospective study conducted on secondary-school adolescents (aged 12–18), has estimated the annual cumulative incidence of pain for each musculoskeletal location and found highest rates for lower limb pain (30%) [8], followed by neck/upper limb pain (28%) [9]. The same authors reported the overall annual incidence of musculoskeletal pain, in all locations, as high as 38% [10]. Similar comprehensive reports in preteens and early adolescent populations are not yet available.

Significant cross-sectional correlations have been reported between childhood musculoskeletal pain and a number of potential risk factors. These include biological/structural factors [11,12]. anthropometric factors [13,14], psychological [15-17] and various life style factors [18,19]. Inconsistent, and sometimes contradictory, cross-sectional findings have also been reported, particularly concerning the predictive role of hypermobility [20,21] and physical exercise [22,23]. In addition, associations in cross-sectional studies cannot always be interpretated as causal due to the problem of establishing the temporal relationship.

A prospective 1-year follow-up study of high-school children showed that psychological rather than physical factors were associated with an increased risk of development of neck/upper limb pain [9] and lower limb pain [10]. Similarly, prospective studies on low back pain conducted so far have provided increasing evidence that psychological and psychosocial factors predict the incidence of low back pain [24] and widespread pain [25] in schoolchildren. However, most of the previously mentioned prospective studies were conducted among mid and/or late adolescents. Studying younger children may provide further insight for understanding of the origin and early contributing factors to chronic musculoskeletal pain in adults. These may be particularly relevant in terms of influence on long-term symptoms.

This study is a continuation of a previous survey of preteens from a general school population. The results on prevalence, outcome and predictive factors of pain persistence have been reported earlier [2,26,27]. We have also previously reported that non-traumatic and traumatic lower limb pains are not similar in terms of risk factors, consequences and long-term prognoses [28,29]. Hence, in this prospective study we followed up subjects who were initially free of musculoskeletal pain, in order to separately estimate occurrence of new-onset non-traumatic and traumatic pains in all musculoskeletal locations and investigate risk factors for development of these symptoms.

## Methods

**Figure 1:**
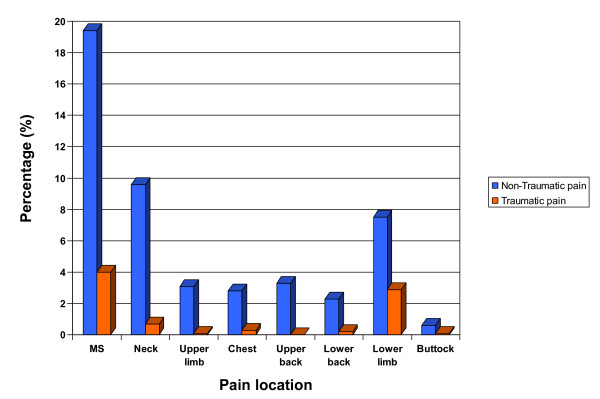
**Development of musculoskeletal pain**. Prevalence of new-onset traumatic and non-traumatic musculoskeletal (MS) pain in schoolchildren in Lahti, Finland. Percentages represent proportions of originally pain-free children who developed musculoskeletal pain at 1-year follow-up.

The study started in March 1995 when all primary schools in Lahti, Finland were asked to take part in a survey to assess pain symptoms in Finnish preteens. Lahti is a town of approximately 95,000 inhabitants (1995) in southern Finland, with 21 primary schools. Two primary schools refused to participate and all pupils from the third and fifth grades, in the remaining 19 schools, and present at school on the day of the survey participated in the study [2,30]. The parents were informed about the study by letters and both the children and their parents had the possibility to refuse enrolment in the survey. The final sample consisted of 1756 children, of whom 867 were third-grade (mean age 9.8 [SD = 0.4] years) and 889 were fifth-grade (mean age 11.8 [SD = 0.4] years) schoolchildren. This sample represented 82.9% of the children of these age groups in Lahti attending normal or special schools (Figure 1). These children completed a pain questionnaire and were tested for hypermobility. Of them 1192 children (68%) did not report pain in any musculoskeletal location with a frequency of at least once per week during the past three months. These children constituted our study population. At 1-year follow-up, 1113 subject (93.4%) were found and re-evaluated to determine the occurrence of new-onset musculoskeletal pain and investigate factors contributing to it. This study was approved by the Ethics Committee of the Health Care Centre of the City of Lahti.

### Instruments

#### A – pain questionnaire

A structured pain questionnaire was designed to assess musculoskeletal pain symptoms (neck, upper limb, chest, upper back, lower back, buttock) during the last three months ('since Christmas'). Musculoskeletal pain symptoms were classified according to pain frequency (pain seldom or never, once a month, once a week, more than once a week, almost daily). The 5-level frequency classification was adopted from the questionnaire used in a nationwide survey on health and health-related behaviours in schoolchildren by the WHO [31]. The questionnaire contained a pain drawing, and the body area concerned was marked in a picture beside the question to help the child to recognize the named area. Children were also asked whether they had directly traumatized the pain areas they had drawn (e.g. injured when exercising, fallen down, stumbled), and those who had experienced direct trauma were asked to mark the pain area with a different colour on the drawing.

The questionnaire also evaluated the occurrence of the following symptoms: 1-headache, 2-abdominal pain, 3-feeling sad or down 4-day-time tiredness, 5-difficulties in falling asleep, and 6-waking up during nights. These symptoms, which we collectively refer to as "psychosomatic", were asked about with the same frequency categorization as musculoskeletal pain, and each of them was considered positive if it had been present at least once per week during the preceding 3 months. The children were asked also about the frequency with which they had exercised to breathlessness, for at least half an hour, during the preceding 3 months and were categorized into 3 groups (0–2, 3–4, and 5–7 times a week). Vigorous exercise was defined as practicing exercise, to breathlessness, for at least half an hour and with a frequency of 5–7 times a week.

During the study design phase, two drafts of the questionnaire were tested in two different schools in Nastola, a neighbouring community of Lahti, Finland and the final draft was evaluated by repeating the survey at an interval of one week. The test-retest reliability of the questionnaire in detecting children with pain at least once per week was good (Kappa (κ) 0.9). The concurrent validity of the pain questionnaire was examined by comparing it with interviews of 31 third- and 25 fifth-grade children. The observed agreement between pain questionnaire and interview technique was 86% (95% confidence interval [95% CI] 74 to 94) and κ was 0.67 [30].

#### B – hypermobility test

A hypermobility test was conducted at baseline using Beighton's method (score 0 to 9) [32]. A nurse, specially trained for the tests, tested the children during school lessons. The intra-and inter-observer reliability had been measured earlier with a kappa coefficients of 0.75 and 0.78 respectively [30]. Out of 1192 pain-free children at baseline, 1142 (95.8%) were examined for hypermobility. School absence on the examination date was the reason for non-participation. A Beighton score of six was chosen as the cut-off point for hypermobility based on the distribution of the results [30].

### Follow-up

The 1-year follow-up was conducted in March 1996. At this follow-up, 1113 (93.3%) of the children who reported no musculoskeletal pain at baseline filled in the same pain questionnaire. Reasons for non-participation in the follow-up assessment included absence from school on the day of testing, changing schools, moving away and refusing to participate. A multivariate logistic regression analysis was conducted to assess the impact of various baseline factors on the odds that a child would be lost to follow-up, and none of the baseline variables significantly influenced the likelihood of dropout.

### Case definition of musculoskeletal pain

At 1-year follow-up, children who reported pain in any musculoskeletal location, uninitiated by a direct trauma, with a frequency of at least once per week during the previous 3 months were classified as having new-onset non-traumatic musculoskeletal pain. On the other hand, those who reported pain in any musculoskeletal location, initiated by a direct trauma, with a frequency of at least once per week during the previous 3 months were classified as having new-onset traumatic musculoskeletal pain.

### Statistical methods

Logistic regression was used to assess the following potential risk factors, age (below/above 11 years), sex, the six psychosomatic symptoms (present/absent), frequency of exercise (0–2/3–4/5–7 times a week) and Beighton score of hypermobility (less than six/six or more). The logistic models were first fitted with each of these variables separately, to estimate the univariate odds ratios (ORs). All independent variables were then included in the multivariate regression equation to estimate the adjusted ORs for each of them. In all logistic regression models, comparisons were performed, with respect to potential risk factors, between children not meeting the case definition of musculoskeletal pain and those with pain symptoms (traumatic and non-traumatic symptoms separately). In all tests, a P value of less than 0.05 (two-tailed) was considered statistically significant. All statistical analyses were performed using SPSS (for Windows), version 10.0.

## Results

### Occurrence of musculoskeletal pain

At one-year follow-up, 239 children (21.5% [95% CI 18.5 to 23.7]) reported new-onset musculoskeletal pain in at least one part of the body. Of them 216 children (19.4% [95% CI 18.5 to 23.4]) reported non-traumatic musculoskeletal pain and 44 children (4.0% [95% CI 3.1–4.6]) had traumatic pain. Twenty-one children reported both types of pain episodes (9.7% of the non-traumatic pain group and 47.7% of the traumatic pain group). The neck was the most commonly reported site with non-traumatic musculoskeletal pain, while the lower limb was the most common site for traumatic pain. Of the 1113 pain-free preteens at baseline, 107 (9.6% [95% CI 7.7 to 11.3]) had non-traumatic neck pain and 32 (2.9% [95% CI 2.2 to 3.4]) had traumatic lower limb pain (Figure 1).

### Risk factors of future non-traumatic musculoskeletal pain

Table 1 shows numbers and percentages of incident musculoskeletal pain by baseline variables. In the univariate analysis, girls had twice the risk of non-traumatic musculoskeletal pain compared to boys. In addition, all psychosomatic symptoms were statistically significant risk factors of pain onset. In the multivariate analysis, the significant independent predictive factors for development of non-traumatic pain were headache (OR = 1.68, [95% CI 1.16–2.44]) and day-time tiredness (OR = 1.53, [95% CI 1.03–2.26]) Furthermore, borderline significance were found for female gender (OR = 1.39 [95% CI 0.99–1.94]) and difficulty in falling asleep (OR = 1.48 [95% CI 0.99–2.23]) (Table 2). Hypermobility was not predictive of future development of non-traumatic musculoskeletal pain. Almost indistinguishable results were found when we reanalysed the data using a cut-off point of Brighton score > or = 4 (data not shown).

### Risk factors of future traumatic musculoskeletal pain

In the univariate analysis, practicing vigorous exercise increased the risk of future traumatic musculoskeletal pain by approximately three folds. Similarly, children who reported day-time tiredness had more than twice the risk of traumatic pain as children who did not feel tired during the school day. In the multivariate analysis the same two factors, vigorous exercise (OR = 3.40 [95% CI 1.39–8.31]) and day-time tiredness (OR = 2.97 [95% CI 1.41–6.26]) were found as independent predictors (Table 3). Hypermobility was not associated with an increased risk of traumatic musculoskeletal pain. Similar insignificant findings were found when we reanalysed the data using a cut-off point of Brighton score > or = 4 (data not shown).

## Discussion

The present study showed that musculoskeletal pain is common in preteens and early adolescents, with 21 percent of schoolchildren, who were pain-free at baseline, reporting new-onset episodes of pain in at least one musculoskeletal site. Development of non-traumatic musculoskeletal pain was predicted by the prior report of psychosomatic symptoms (including, but not limited to, day tiredness), while development of traumatic pain was predicted by the previous report of day tiredness and practicing vigorous exercise.

We have gathered information on pain experience using a previously validated structured questionnaire, which included a body map to help the child recognise the exact site of musculoskeletal pain experience. Self-report tools, the 'gold standard ' for assessing children's pain, are appropriate for children 4 years and older and provide the most reliable and valid approach to measuring pain [33]. School-aged children are also able to be very specific in locating pain [34]. Current evidence indicates that using a variety of tools in assessing childhood pain would provide a richer description of pain experience. However, we have only used one tool (pain questionnaire) to evaluate musculoskeletal pain. In addition, we did not evaluate pain intensity. It is possible that psychological factors or other aspects of the children's personality could have influenced their pain reports. However "psychogenic pain", which is neither well defined nor accurately measured [35] and cannot be separated from somatic pain in population-based epidemiological studies. Several possible risk factors of childhood musculoskeletal pain were not assessed, these include several biological, socio-demographic and anthropometric factors. However, it must be noted that the main aim of this study was to investigate easily measured risk factors of musculoskeletal pain in preteens and early adolescents and identify high risk groups for primary prevention purposes. Another methodological factor regarding our study that need to be addressed is our reliance on self-completed questionnaire for collecting information on physical activity, rather than the more-valid objective tools (e.g. accelerometer and pedometer). Wedderkopp et al conducted a cross-sectional study of 481 Danish schoolchildren and documented a weak correlation between accelerometer measurements and self-reported physical activity [36]. In another recent study in younger children, Hands et al found that physical activity was more accurately measured by the pedometer compared to the accelerometer [37]. Objective data is hard to collect in large population-based studies like ours, and at present there is no generally accepted objective evaluation method to measure physical activity in children.

In this study, we have used a rather strict case definition of pain, with a frequency of at least once a week, for a relatively short recall period (during the previous 3 months) in order to avoid childhood recall difficulties, which have been previously documented [14,38,39]. By using these case definitions of musculoskeletal pain symptoms, we aimed to improve the specificity of the questionnaire in identifying those who developed musculoskeletal pain at 1-year follow-up. We have also separately calculated the incidence of traumatic and non-traumatic pain symptoms. All of the previously mentioned factors limit the comparability of our estimates with those of previous longitudinal studies, which have mostly used a longer pain recall period (6 month or 1 year) and included both traumatic and non-traumatic symptoms in the same case definition. Furthermore, the study populations of almost all previous studies were older than ours (which is composed mainly of children aged 10 and 12 years at baseline). This might partially explain the considerably higher incidence of regional musculoskeletal pain symptoms reported in previous studies compared to ours, given the strong positive association between age and musculoskeletal pain in children and adolescents [5,18,19]. That becomes evident when we compare our site-specific incidence figures with those of Ehrmann-Feldmann et al. cohort study of schoolchildren aged 13–15 years [10]. In their prospective Canadian study, the 6-month cumulative incidence of lower limb pain, upper limb pain, low back pain (all in a frequency of at least once a week) were estimated to be 22%, 20%, 13%, respectively. All these site-specific figures were 2–3 times higher than those found in our study population. Despite these differences, yet the overall incidence of all musculoskeletal pain in our preteen/early-adolescent study subjects (21.5%) was just slightly lower than that found in Ehrmann-Feldmann et al's study of middle-adolescent subjects (27.1%) [10]. This might indicate that co-occurrence of different localized musculoskeletal pain symptoms is more commonly reported by middle-adolescents than preteens and early-adolescents. A finding, which is in line with our previous observation that musculoskeletal pain tends to be more generalized as the child grows up [27]. It must be noted that, unlike other site-specific musculoskeletal pain symptoms, the incidence proportion of neck pain in our study population (9.4%) is comparable to that found in the previously mentioned Canadian study (10%) [10]. This might indicate that neck pain, unlike other musculoskeletal pain symptoms, follows a steady pattern of incidence increase with age and that this trend starts as early as preadolescence. This pattern is different from low back pain, for example, where there is a steep increase of reporting at adolescence with an annual incidence almost doubled between the ages 12 and 15 years olds [5].

In the present study, 4.0% of schoolchildren who were pain-free at baseline reported traumatic musculoskeletal pain at 1-year follow-up. Although our questionnaire did not address the cause of the trauma, we assume that most injuries were related to sports and with this assumption our results goes well with previous studies reporting that between 3% and 11% of schoolchildren experience exercise-related injuries annually [40]. We also found that lower limb was the most common area with traumatic pain, which is similar to a previous report on children at an athletic setting [41].

In the current study, girls had approximately 40% higher risk for development of non-traumatic musculoskeletal pain compared to boys. Although this association was only border-line statistically significant in the multivariate analysis, it is unlikely that this association is only a chance finding, given the narrow confidence interval and the strong evidence in the majority, but not all, of previous studies [42]. Similarly, significant associations were found between incident non-traumatic musculoskeletal pain and all psychosomatic symptoms, and independent correlations were found between non-traumatic pain with both headache and day-time tiredness. This is in accordance with earlier reports that documented the predictive role of headache on future low back pain in adolescents [7], the evident association between day-time tiredness and persistence of musculoskeletal pain in children [26], and the strong correlation between fatigue and chronic pain syndromes, such as fibromyalgia [43]. It must be noted that we have used the term "psychosomatic" to refer to symptoms that are chiefly considered expressions of psychological stress rather than manifestations of organic disorders based on previous research in children [44,45]. The same term was used in our previous reports [26,27], as well as by other researchers [46,47].

We identified two risk factors of trauma-induced pain: vigorous exercise and day-time tiredness. Both factors had an independent role in pain development. The role of vigorous exercise on occurrence of trauma-induced musculoskeletal pain in adolescents is supported both by previous studies [48,49], and by logic (the more the child participates in sports, the more chance of being injured). The strong role of day-tiredness on development of traumatic musculoskeletal can not be explained by the confounding effect of vigorous exercise, as day-time tiredness independently predicted the onset of traumatic pain in the multivariate analysis (i.e. after adjusting for the effect of exercise frequency). One possible explanation would be that children with day-time tiredness are exhausted, fatigued and un-able to adjust their movement or posture to protect themselves from being injured in sports fields-regardless of the frequency of exercise performed- and possibly in other settings. This assumption is in accordance with a previous report identifying "overtiredness" as a one of the contributing factors to sports injuries [50].

Occurrence of new-onset traumatic and non-traumatic musculoskeletal pain did not differ between children with and without hypermobility. This insignificant association is similar to our earlier finding [26,30], and in accordance with another Nordic study in preteens [1]. However, most previous cross-sectional studies have reported a clear association between hypermobility and arthralgia in children [32,51], and recently we have identified hypermobility as a strong contributing factor for long-term persistence of musculoskeletal pain in children [27,29]. Obviously, more studies are still warranted to unveil the role of hypermobility in childhood musculoskeletal pain.

## Conclusion

Our results suggest that nearly one fifth of preteens and early adolescents experience new-onset weekly musculoskeletal pain over a one-year period. The neck is the most common site of non-traumatic pain, while the lower limb is the most common site of traumatic pain. Psychosomatic symptoms, rather than physical factors, predict future non-traumatic pain, while both physical and psychosomatic factors predict future traumatic pain. This study highlights that there may be two types of pain entities with both distinct and common aspects of aetiology. For primary prevention purposes, school healthcare professionals should pay attention to preteens and early adolescents practicing vigorous exercise (predictor of traumatic pain), reporting headache (predictor of non-traumatic pain) and reporting day-time tiredness (predictor of both types of pain).

## Declaration of interests

The author(s) declare that they have no competing interests.

## Authors' contributions

Dr. Ashraf El Metwally had primary responsibility for data analysis, and writing the manuscript. Professor Jouko Salminen supervised the design of the study and contributed to the writing of the manuscript. Professors Anssi Auvinen and Gary Macfarlane participated in the analytic framework for the study, and contributed to the writing of the manuscript. Dr. Marja Mikkelsson developed the protocol and questionnaire, designed the study, supervised and participated in data collection, and contributed to the writing of the manuscript. All authors read and approved the final manuscript.

## Pre-publication history

The pre-publication history for this paper can be accessed here:



## Figures and Tables

**Table 1:** Numbers and proportions of new-onset traumatic and non-traumatic musculoskeletal pain in 1113 follow-up responders free of musculoskeletal pain at baseline, stratified by baseline factors (Lahti schoolchildren population sample, 1995–1996).

Variables	Category	Total number	Non-traumatic musculoskeletal pain, N (%)	Traumatic musculoskeletal pain, N (%)
Sex	Male	542	86 (15.9)	24 (4.4)
	Female	571	130 (22.8)	20 (3.5)
Age	9–10 years	538	99 (18.4)	22 (4.1)
	11–13 years	575	117 (20.3)	22 (3.8)
°Headache	Yes	228	66 (29.0)	12 (5.3)
	No	867	150 (17.3)	31 (3.6)
°Abdominal pain	Yes	118	37 (31.4)	5 (4.2)
	No	980	178 (18.2)	38 (3.9)
°Feeling sad/down	Yes	126	38 (30.2)	6 (4.8)
	No	963	174 (18.1)	37 (3.8)
°Difficulty falling asleep	Yes	203	61 (30.0)	12 (5.9)
	No	894	150 (16.8)	31 (3.5)
°Day-time tiredness	Yes	245	73 (29.8)	17 (6.9)
	No	856	140 (16.4)	26 (3.0)
°Waking up during nights	Yes	135	38 (28.1)	9 (6.7)
	No	959	174 (18.1)	34 (3.5)
^a^Hypermobility score	Less than 6	83	13 (15.7)	2 (2.4)
	6 or more	983	193 (19.6)	39 (4.0)
Frequency of exercise	0–2 times a week	587	114 (19.4)	16 (2.7)
	3–4 times a week	342	65 (19.0)	14 (4.1)
	5–7 times a week	150	32 (21.3)	12 (8.0)

°Symptoms reported in a frequency of at least once a week.

^a^Using Beighton test. A child was categorized as hypermobile if his/her hypermobility score was six or more.

**Table 2:** Odds ratios and confidence intervals for future non-traumatic musculoskeletal pain at 1-year follow-up by baseline predictive factors, using univariate and multivariate logistic regression analyses (1113 preteens and early adolescents).

Variables	Non-traumatic musculoskeletal pain Univariate analysis	Non-traumatic musculoskeletal pain Multivariate analysis
Age		
9–10 years	Reference	Reference
11–13 years	1.13 (0.84–1.53)	1.07 (0.77–1.48)
Sex		
Males	Reference	Reference
Females	1.56 (1.16–2.11)	1.39 (0.99–1.94)
Psychosomatic symptoms^1^		
Headache	1.95 (1.39–2.73)	1.68 (1.16–2.44)
Abdominal pain	2.06 (1.35–3.14)	1.40 (0.85–2.30)
Feeling sad or down	1.96 (1.29–2.96)	1.18 (0.73–1.93)
Difficulties in falling asleep	2.13 (1.51–3.02)	1.48 (0.99–2.23)
Day-time tiredness	2.16 (1.56–3.01)	1.53 (1.03–2.26)
Waking up during nights	1.77 (1.17–2.66)	1.31 (0.82–2.08)
Hypermobility score°		
Less than 6	Reference	Reference
6 or more	0.76 (0.41–1.41)	0.83 (0.44–1.56)
Frequency of physical exercise		
0–2	Reference	Reference
3–4	0.97 (0.69–1.37)	1.07 (0.74–1.54)
5–7	1.13 (0.72–1.75)	1.38 (0.85–2.48)

^1^Reported in a frequency of at least once a week

°Using Beighton test. A child was categorized as hypermobile if his/her hypermobility score was six or more.

**Table 3:** Odds ratios and confidence intervals for future traumatic musculoskeletal pain at 1-year follow-up by baseline predictive factors, using univariate and multivariate logistic regression analyses (1113 preteens and early adolescents).

Variables	Traumatic musculoskeletal pain Univariate analysis	Traumatic musculoskeletal pain Multivariate analysis
Age		
9–10 years	Reference	Reference
11–13 years	0.93 (0.51–1.71)	0.64 (0.32–1.26)
Sex		
Males	Reference	Reference
Females	0.78 (0.43–1.44)	0.90 (0.45–1.81)
Psychosomatic symptoms^1^		
Headache	1.50 (0.76–3.00)	1.22 (0.56–2.65)
Abdominal pain	1.10 (0.42–2.84)	0.85 (0.30–2.41)
Feeling sad or down	1.25 (0.52–3.03)	0.80 (0.30–2.14)
Difficulties in falling asleep	1.75 (0.88–3.47)	1.47 (0.67–3.24)
Day-time tiredness	2.37 (1.27–4.45)	2.97 (1.41–6.26)
Waking up during nights	1.94 (0.91–4.15)	1.64 (0.70–3.85)
Hypermobility score°		
Less than 6	Reference	Reference
6 or more	0.60 (0.14–2.52)	0.70 (0.16–3.04)
Frequency of physical exercise		
0–2	Reference	Reference
3–4	1.52 (0.73–3.16)	1.80 (0.83–3.93)
5–7	3.10 (1.44–6.71)	3.40 (1.39–8.31)

^1^Reported in a frequency of at least once a week

°Using Beighton test. A child was categorized as hypermobile if his/her hypermobility score was six or more.
